# Myelin Oligodendrocyte Glycoprotein Antibody Disease After COVID-19 Vaccination - Causal or Incidental?

**DOI:** 10.7759/cureus.27024

**Published:** 2022-07-19

**Authors:** Vineet Sehgal, Priyanshu Bansal, Shaifali Arora, Saniya Kapila, Gaganpreet S Bedi

**Affiliations:** 1 Neurology, Amandeep Medicity, Amritsar, IND; 2 Neurology, Sehgal's Neuro and Child Care Centre, Amritsar, IND; 3 Medicine, Fortis Escorts Hospital, Amritsar, Amritsar, IND; 4 Radiology, Amandeep Medicity, Amritsar, IND

**Keywords:** chadox1 ncov-19, demyelination syndrome, anti-mog antibody, mogad, covid-19

## Abstract

A variety of neurological complications of COVID-19 vaccinations are reported with each passing day. We report a rare case of a patient who developed brain stem and spinal cord lesions 20 days after the first dose of the ChAdOx1 nCoV-19 coronavirus vaccine. On further investigation, anti-myelin oligodendrocyte glycoprotein (anti-MOG) antibodies were strongly positive, and a diagnosis of myelin oligodendrocyte glycoprotein antibody disease (MOGAD) was made. This case report aims to add a rare entity to the possible complications of COVID-19 vaccines and discuss symptoms, MRI findings, and the differential diagnosis of MOGAD. We also suggest that these patients undergo follow-up serological tests to recognize recurrences quickly.

## Introduction

Myelin oligodendrocyte glycoprotein antibody disease (MOGAD) consists of symptoms resulting from a demyelinating inflammatory process involving the central nervous system. With an immune-mediated attack mediated against the myelin oligodendrocyte glycoprotein, the MOGAD can affect the optic nerve, the brain, and the spinal cord [1]. The resulting signs and symptoms include, but are not limited to, optic neuritis with decreased visual acuity, acute disseminated encephalomyelitis (ADEM), brainstem attacks, and transverse myelitis. We are reporting a case of myelin oligodendrocyte glycoprotein antibody disease, which occurred 20 days after the patient was given the first dose of the ChAdOx1nCoV-19 coronavirus vaccine.

## Case presentation

A 26-year-old male presented to emergency with chief complaints of progressive bilateral upper and lower limb weakness for the past two days. On the third day of illness, the patient complained of urinary retention. He had a reduced sensation of pain and touch below the level of C4. No history of diplopia, decreased visual acuity, difficulty in swallowing, chewing, and breathing difficulty. The patient gave a history of his first dose of ChAdOx1 nCoV-19 coronavirus vaccination 20 days before all his symptoms started. He had never had any similar episode in the past. There was no history suggestive of connective tissue disorder, viral illness, trauma, or dog bite in the past.

On examination, the patient had complete paralysis of both upper and lower limbs. The power was grade 2/5 proximally and grade 3/5 distally in both upper and lower limbs), with diminished touch, pain, and vibration below C4 dermatome level. In addition, the patient had urinary retention, for which catheterization was done. All reflexes were brisk, and Babinski's reflex was extensor.

**Figure 1:**
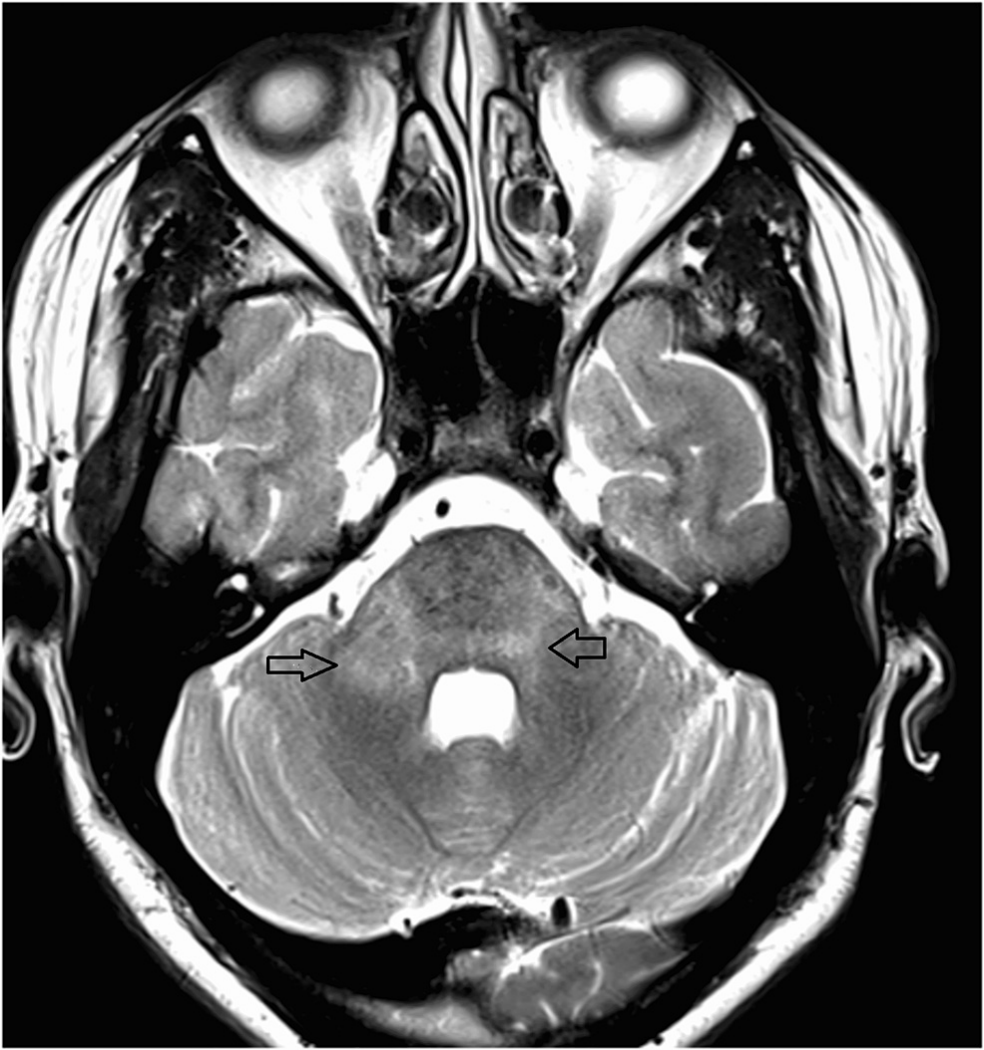
Hyperintense areas are seen in the pons and both middle cerebellar peduncles (RT >LT) on axial T2W images RT - right, LT - left

**Figure 2:**
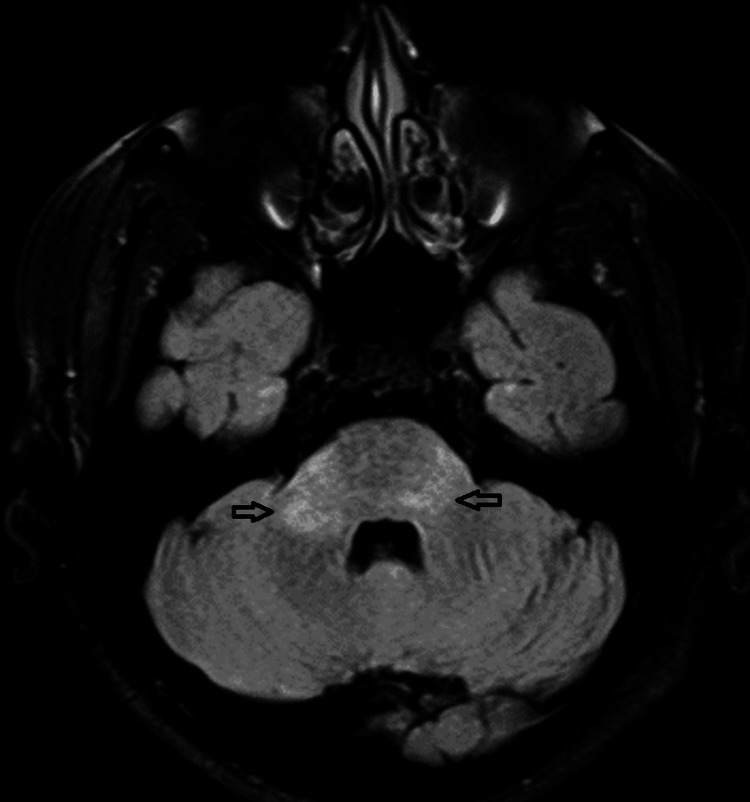
Hyperintense areas are seen in the pons and both middle cerebellar peduncles (RT >LT) on FLAIR images FLAIR - fluid attenuated inversion recovery, RT - right, LT - left

**Figure 3:**
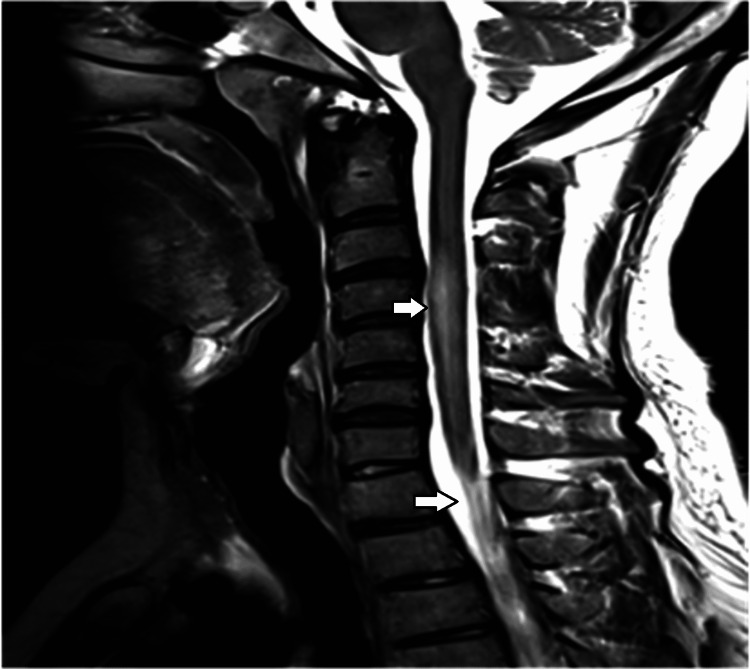
Long segment of hyperintense signal C3-C6 is seen in the cervical cord on sagittal T2W images

MRI of the brain and spine was performed. It showed hyperintensities in bilateral middle cerebellar peduncles and pons (Figure 1, 2) and hyperintense signals from C3 to C6 levels in the spine (Figure 3). None of the lesions showed contrast enhancement.

The possibilities of acute disseminated encephalomyelitis (ADEM), neuromyelitis optica spectrum disorder (NMOSD), multiple sclerosis (MS), myelin oligodendrocyte glycoprotein antibody disease, post-viral demyelination, post vaccinal demyelination, connective tissue disorders like systemic lupus erythematosus (SLE), vasculitis, and neuro-sarcoidosis were considered.

General physical examination was unremarkable. Serum venereal disease research laboratory (VDRL), antinuclear antibody (ANA), cytoplasmic antineutrophil cytoplasmic antibodies (C-ANCA), perinuclear antineutrophil cytoplasmic antibody (P-ANCA), hepatitis B, hepatitis C, and HIV were negative, and angiotensin-converting enzyme (ACE) levels were normal. Cerebrospinal fluid (CSF) examination showed a raised lymphocytic count of 184/mm^3^ with a slight increase in protein level - 88mg/dl. The examination for oligoclonal bands in CSF, the pan neurotropic viral panel, and the Bio-fire panel was negative for all common bacteria, viruses, and fungi. Similarly, all stains, cultures, and malignant cells were negative. Aquaporin-4 antibodies were negative in both CSF and serum. IgG anti-MOG antibodies were strongly positive in serum. Evoked potentials like VER and BAER were within normal limits. Levels of anti-spike protein COVID-19 antibodies were elevated. Reverse transcript (RT) COVID-19 polymerase chain reaction (PCR) was negative in the nasopharyngeal swab. 

The absence of oligoclonal bands, MRI pictures, and CSF findings with strongly positive IgG anti-MOG antibodies points toward anti-MOG antibody syndrome. Also, the presence of anti-spike protein COVID-19 antibodies and recent administration of vaccine hints toward a possibility of vaccine-associated MOGAD.

**Figure 4:**
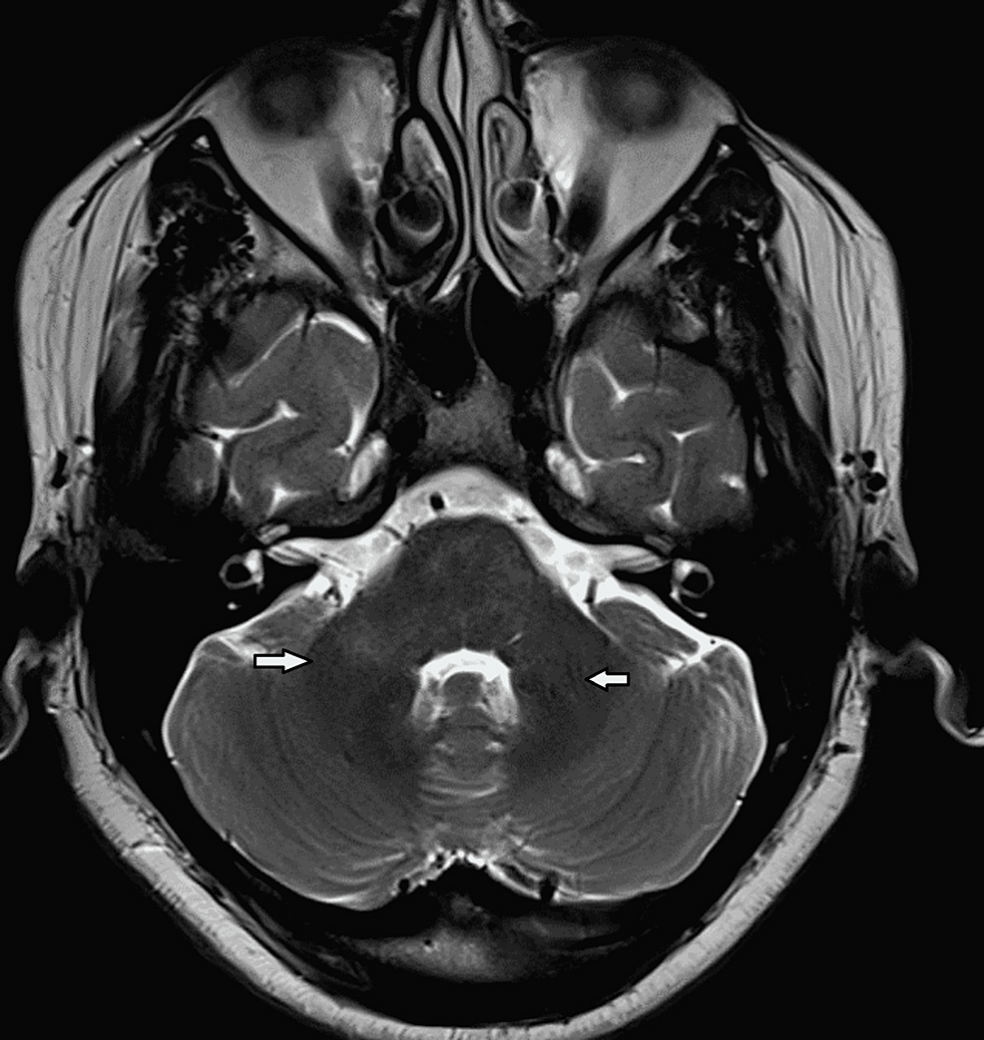
Marked resolution of hyperintense areas is seen in pons and adjacent bilateral middle cerebellar peduncles on axial T2W images after eight weeks of steroids

**Figure 5:**
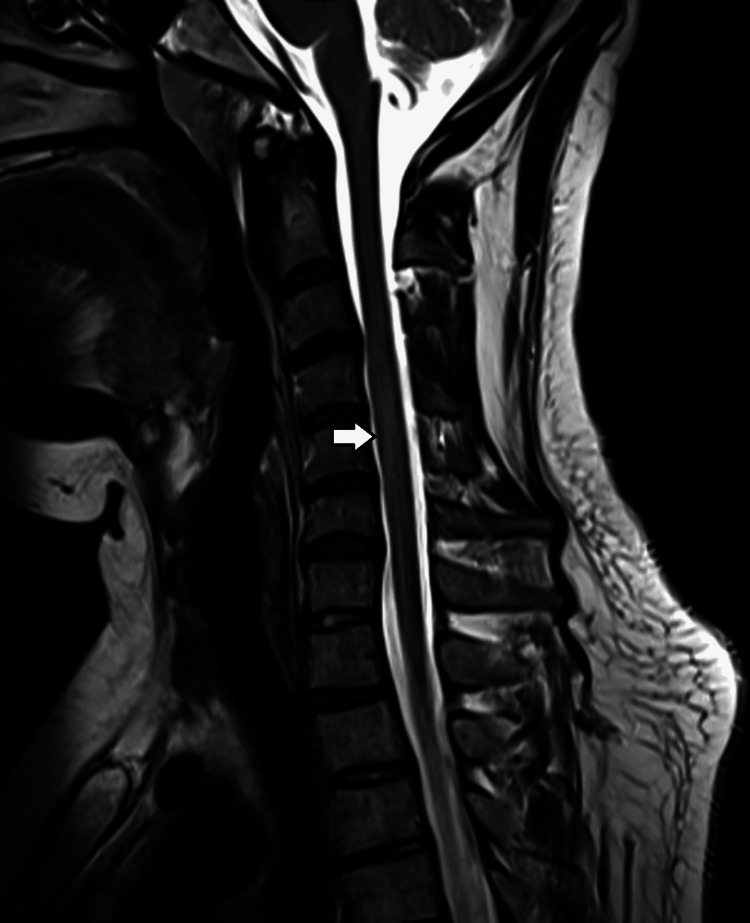
Marked resolution of hyperintense areas is seen in the spinal cord on sagittal T2W images after eight weeks of steroids

The patient was treated with 1 gm I/v once daily methylprednisolone for five days, following which he started showing signs of recovery on the fifth day, followed by tapering dosages of oral steroids over eight weeks. Within a month, his strength in the upper and lower limbs has improved, and after a gap of four weeks, he has started walking independently and regained control of his bowel and bladder. Sensory loss has gradually improved over the next few weeks. His MRI of the brain and spine after eight weeks show significant resolution of lesions (Figure 4, 5). 

## Discussion

Similar to the rise of immune-mediated neurological diseases amidst the coronavirus pandemic [1,2], cases of myelin oligodendrocyte glycoprotein antibody disease (MOGAD) are increasingly being reported in association with the COVID-19 virus [3] and its vaccination. MOGAD consists of a spectrum of symptoms that can involve the optic nerve, brain, brainstem, and spinal cord in various combinations. Therefore, the patient can present as an isolated case of optic neuritis or myelitis, acute demyelinating encephalomyelitis, encephalitis, or any possible combination [4,5,6,7}. Similar to our case, various authors have described MOGAD after ChAdOx1 nCoV-19 coronavirus vaccination leading to myelitis, ADEM, brainstem encephalitis, encephalitis-like presentation [1,2,4-9]. Furthermore, other vaccines like influenza and human papillomavirus are frequently linked to central nervous system demyelination [10]. However, the incidence of post-COVID-19 vaccinal myelitis is very low, and the benefit of preventing serious infections outweighs the risk of the rare postvaccination events [11]. 

Our patient presented as a case of acute demyelinating encephalomyelitis-like condition with hyperintensities in bilateral middle cerebellar peduncles, pons, and the hyperintense signal from C3 to C6 levels as seen on brain and spine MRI after 20 days of vaccination. The long-segment myelitis, brain MRI findings, positive anti-MOG-IgG antibodies, absence of any other etiologies, and history of COVID-19 vaccination 20 days back favor the diagnosis of MOGAD. However, it is essential to differentiate it from the other neurological demyelinating disorders associated with the COVID-19 vaccine, like neuromyelitis optica spectrum disorder (NMOSD), post-vaccinal myelitis, and acute attack of multiple sclerosis triggered by the vaccine. The clinical signs and symptoms, brain and spine imaging, CSF findings, and serology help differentiate MOGAD from Multiple sclerosis [12,13] and anti-aquaporin-4 antibody-related demyelination [14]. In MOGAD, the involvement of the optic nerve can be unilateral or bilateral, and the anterior visual pathway is extensively affected. With anti-aquaporin-4 related demyelinating disorder [14], the optic nerve involvement can also be unilateral or bilateral; however, the posterior optic pathway is majorly involved, including the optic chiasm. The long-segment myelitis and the positive AQP4-IgG hint toward its diagnosis. With multiple sclerosis [12,13], the classic presentation involves unilateral optic neuritis, short segment myelitis, and oligoclonal bands in CSF in about 80-85% of the patients. Optic nerve MRI in multiple sclerosis shows the enhancement of the middle segment of the optic nerve (less than 50% of the length of the nerve) [15]. On MRI brain thalamic, pontine lesions are more common in MOGAD than AQP4-positive disease. Compared to AQP4-positive patients, cerebellar peduncle lesions are only found in anti-MOG antibody-positive children. In children, both short and long segment lesions have been described in MOGAD [16].

Our patient received his first dose of COVID-19 vaccine 20 days before being tested positive for the anti-MOG antibody. However, it could not be established whether the development of MOGAD was linked to the recent vaccination or the detection was purely incidental. It is a known fact that vaccines can not only trigger monophasic demyelinating disorder but can unmask chronic relapsing disorder [8]. Therefore, we plan to follow up with the patient over the next couple of months and repeat the serology at serial intervals to check for the presence or absence of anti-MOG antibodies as it has therapeutic and prognostic implications. The continuous presence of anti-MOG antibodies is associated with relapse, which is seen in 44-83% [16] of patients with MOG-IgG-associated demyelinating disorders, and such patients require long-term immunosuppressive therapy. The relapsing course was more likely present in patients with higher MOG-IgG titers at onset and persisting MOG-IgG over time. In contrast, transient low titer MOG-IgG was typically associated with a monophasic disease course [17]. There is no definite consensus regarding the monitoring of antibodies. However, some authors have suggested that a 6-12 months re-test interval may be helpful [18]. 

With emerging data worldwide, there seems to be a temporal link between these two; however, a causal relationship has not been identified, and more studies and research have to be conducted at a population level. If established, possible mechanisms include molecular mimicry (cross-reaction between vaccine antigens and myelin proteins), expansion and stimulation of autoreactive T-cell clones, enhanced antigen presentation, and epitope spreading, which could trigger CNS demyelination [19]. 

## Conclusions

This case report aims to shed light on myelin oligodendrocyte glycoprotein antibody disease, another stratum of neurological complications seen with COVID-19 vaccination, to aid in early diagnosis and treatment. It is unknown whether MOGAD is an incidental finding or has any temporal relation with the COVID-19 vaccine. We suggest that patients like this should get a follow serology test to manage therapeutic and prognostic implications. We want to affirm that this association, by no means, warrants the avoidance of vaccination. There is no data to suggest that any neurological condition is a contraindication for COVID-19 vaccination, as its benefits far outweigh the concerns.

## References

[1] Fernandes J, Jaggernauth S, Ramnarine V, Mohammed SR, Khan C, Panday A (2022). Neurological conditions following COVID-19 vaccinations: chance or association?. Cureus.

[2] Goss AL, Samudralwar RD, Das RR, Nath A (2021). ANA investigates: neurological complications of COVID-19 vaccines. Ann Neurol.

[3] Peters J, Alhasan S, Vogels CB, Grubaugh ND, Farhadian S, Longbrake EE (2021). MOG-associated encephalitis following SARS-COV-2 infection. Mult Scler Relat Disord.

[4] Mumoli L, Vescio V, Pirritano D, Russo E, Bosco D (2022). ADEM anti-MOG antibody-positive after SARS-CoV2 vaccination. Neurol Sci.

[5] Escolà JK, Deuschl C, Junker A (2022). MOG antibody-associated encephalomyelitis mimicking bacterial meningomyelitis following ChAdOx1 nCoV-19 vaccination: a case report. Ther Adv Neurol Disord.

[6] Zhou S, Jones-Lopez EC, Soneji DJ, Azevedo CJ, Patel VR (2020). Myelin oligodendrocyte glycoprotein antibody-associated optic neuritis and myelitis in COVID-19. J Neuroophthalmol.

[7] Nagaratnam SA, Ferdi AC, Leaney J, Lee RL, Hwang YT, Heard R (2022). Acute disseminated encephalomyelitis with bilateral optic neuritis following ChAdOx1 COVID-19 vaccination. BMC Neurol.

[8] Kumar N, Graven K, Joseph NI, Johnson J, Fulton S, Hostoffer R, Abboud H (2020). Case report: postvaccination anti-myelin oligodendrocyte glycoprotein neuromyelitis optica spectrum disorder: a case report and literature review of postvaccination demyelination. Int J MS Care.

[9] Jarius S, Ruprecht K, Kleiter I (2016). MOG-IgG in NMO and related disorders: a multicenter study of 50 patients. Part 2: epidemiology, clinical presentation, radiological and laboratory features, treatment responses, and long-term outcome. J Neuroinflammation.

[10] Karussis D, Petrou P (2014). The spectrum of post-vaccination inflammatory CNS demyelinating syndromes. Autoimmun Rev.

[11] Malhotra HS, Gupta P, Prabhu V, Kumar Garg R, Dandu H, Agarwal V (2021). COVID-19 vaccination-associated myelitis. QJM.

[12] Havla J, Schultz Y, Zimmermann H, Hohlfeld R, Danek A, Kümpfel T (2022). First manifestation of multiple sclerosis after immunization with the Pfizer-BioNTech COVID-19 vaccine. J Neurol.

[13] Toljan K, Amin M, Kunchok A, Ontaneda D (2022). New diagnosis of multiple sclerosis in the setting of mRNA COVID-19 vaccine exposure. J Neuroimmunol.

[14] Badrawi N, Kumar N, Albastaki U (2021). Post COVID-19 vaccination neuromyelitis optica spectrum disorder: case report & MRI findings. Radiol Case Rep.

[15] Flanagan EP (2019). Neuromyelitis optica spectrum disorder and other non-multiple sclerosis central nervous system inflammatory diseases. Continuum (Minneap Minn).

[16] Wynford-Thomas R, Jacob A, Tomassini V (2019). Neurological update: MOG antibody disease. J Neurol.

[17] Hegen H, Reindl M (2020). Recent developments in MOG-IgG associated neurological disorders. Ther Adv Neurol Disord.

[18] Jarius S, Paul F, Aktas O (2018). MOG encephalomyelitis: international recommendations on diagnosis and antibody testing. J Neuroinflammation.

[19] Langer-Gould A, Qian L, Tartof SY (2014). Vaccines and the risk of multiple sclerosis and other central nervous system demyelinating diseases. JAMA Neurol.

